# Retraction: Highly sensitive cadmium sulphide quantum dots as a fluorescent probe for estimation of doripenem in real human plasma: application to pharmacokinetic study

**DOI:** 10.1039/d4ra90134g

**Published:** 2024-11-11

**Authors:** Marwa F. B. Ali, Baher I. Salman, Samiha A. Hussein, Mostafa A. Marzouq

**Affiliations:** a Department of Pharmaceutical Analytical Chemistry, Faculty of Pharmacy, Assiut University Assiut 71526 Egypt; b Department of Pharmaceutical Analytical Chemistry, Faculty of Pharmacy, Al-Azhar University Assiut Branch Assiut 71524 Egypt bahersalman2013@yahoo.com bahersalman@azhar.edu.eg +201099031345

## Abstract

Retraction of ‘Highly sensitive cadmium sulphide quantum dots as a fluorescent probe for estimation of doripenem in real human plasma: application to pharmacokinetic study’ by Marwa F. B. Ali *et al.*, *RSC Adv.*, 2020, **10**, 44058–44065, https://doi.org/10.1039/D0RA07960J.

The Royal Society of Chemistry, with the agreement of the authors below, hereby wholly retracts this *RSC Advances* article due to concerns with the reliability of the data.

The TEM image in the right panel of Fig 2a shows repeating patterns and inconsistencies in the background that the authors have not been able to satisfactorily explain.

The XRD data in Fig. 3a contain repeating sections.

An independent expert was consulted who was not satisfied with the explanation provided by the authors.

Given the significance of the concern about the validity of the data, the findings presented in this paper are no longer reliable. The authors have cooperated throughout the investigation and have endeavoured to be transparent regarding the errors in the data.

Samiha A. Hussein and Mostafa A. Marzouq were contacted but did not respond.

Signed: Baher I. Salman, Marwa F. B. Ali

Date: 2nd November 2024.

Retraction endorsed by Laura Fisher, Executive Editor, *RSC Advances*

